# Editorial: Exploring the key targets and compounds that manipulate brain neurocircuits against mental disorders and psychiatric

**DOI:** 10.3389/fphar.2024.1411528

**Published:** 2024-04-29

**Authors:** Weijie Xie, Song Zhang

**Affiliations:** ^1^ Shanghai Pudong New Area Mental Health Center, Tongji University School of Medicine, Shanghai, China; ^2^ Renji Hospital, School of Medicine, Shanghai Jiao Tong University, Shanghai, China

**Keywords:** neuropharmachology, neurocircuit, targets, brian network, molecular pathway

Neuropharmacology investigates how drugs influence the nervous system, with the goal of creating medications that can help individuals suffering from psychiatric and neurological disorders ranging from depression and anxiety to schizophrenia and chronic pain. However, neurons in the CNS do not operate in isolation; instead, they work within synaptically connected neural circuits and networks. With advances in neuroscience methods, such as genetic techniques, viral recombination, and *in vivo* imaging, scientists have been able to uncover the neural circuits that underlie neurological and psychiatric diseases. Furthermore, recent studies in *Science* and *Neuron* (Wang et al., 2020; Trieu et al., 2022) have provided a novel insight into the precise regulation of the neural circuit and brain network functions by small molecular compounds targeting the enriched molecules or receptors of these neural circuits, thereby treating psychiatric and neurological diseases. In this Research Topic, we present a collection of original research works from both basic and clinical researchers and reviews that explore the interactions between neural circuits and neuropharmacological approaches to intervene in psychiatric and neurological disorders.

Natural products and compounds are vital resources of exploring new treatments for mental disorders (Zhu et al., 2024). In this Research Topic entitled “*Exploring the Key Targets and Compounds That Manipulate Brain Neurocircuits Against Mental Disorders and Psychiatric*”, there are four original research works that demonstrate the protective effects and therapeutic value for stress-induced psychiatric symptoms, including bioactive compound ginsenosides Rb1 and Rg1 (Jiang et al.), Fraxetin (Ahmed et al.), fresh *Gastrodia* products (Zhang et al.) and traditional Chinese drugs called *Chaihu-jia-Longgu-Muli-tang* (CLM, Zhao et al.). Herein, Rb1 and Rg1 significantly improve the impairment of spatial and associative learning and memory caused by microgravity-induced learning and memory dysfunction in a rat model of hindlimb suspension; administration of fraxetin can ameliorate chronic unpredictable stress-induced behavioral deficits and inhibit the increased corticosterone level; the use of Gastrodia noticeably reverses hindlimb unloading weightlessness-induced cognitive impairment. In additions, the data analysis of twenty-four RCTs shows that CLM treatment could improve the symptoms of anxiety and insomnia. These positive effects of nature products are involved in the inhibition of oxidative stress and apoptosis, increase of synaptic plasticity and regulation of inflammatory factors. The molecular mechanisms involved might be related to the BDNF-TrkB, PI3K-Akt, MAPK, and NF-κB pathways, offering intriguing and insightful approaches for the prevention and treatment of mental disorders.

In contrast to traditional molecular pharmacology, which focuses on the modulatory effects of drug-target interactions, recent advancements in neuroscience have enabled manipulations at the level of brain neurocircuits and networks, allowing for more precise modulation of neurological and psychiatric disorders. The Research Topic section studies show that chronic stress induces the alteration of melatonin receptors and the circadian rhythm, presenting the sleep disorder-related symptoms (Xia et al.). In the nucleus accumbens, the dynamics and kinetics of dopamine release vary distinctly in response to different nicotine salts (Li et al.). In addition to the receptor and neurons, the optogenetic and chemogenetic manipulations of the medial septum (MS)→lateral hypothalamus (LH) glutamatergic projections suppress pain thresholds in the chronic constriction injury model, indicating MS glutamatergic neurons modulate nociception via the projections to LH (Fan et al.). Furthermore, another review dissects the roles of afferent and efferent projections of the rostral ventromedial medulla (RVM) and related neurotransmitters in pain modulation (Peng et al.). However, where to pharmacologically intervene at a larger scale of the brain neurocircuit and network is a common dilemma in the fields of neuropharmacology.

In this Research Topic, the included four clinical trials also provide some novel finding and clues between the molecular targets and clinical phenotypes. For instance, baseline BMI shows a significant correlation with the symptom improvements in schizophrenia (SZ, Chen et al.); cognitive improvement is associated with changes of carnitine metabolite levels after olanzapine monotherapy in CS (Zhao et al.). The clinical neuromodulation data further reveal some therapeutic effects (Zhu et al.) and potential biomarkers (Li et al.) for clinical psychiatrical assessment. In additions, the included two reviews further discuss and explore the dysregulation of copper (Chen et al.) and iron (Sousa et al.) behind neuropsychiatric deficits. These suggest that much work remains to be done in uncovering the neurocircuitry mechanisms and molecular targets underpin the clinical phenotypes of neuropsychiatric disorders in the future.

Overall, our collection of articles highlights the remarkable progress made in identifying potential compounds or natural products and key targets within brain neurocircuits that, when manipulated, offer new hope for individuals battling psychiatric and neurological disorders. Thus, the neurocircuit-regulatory pharmacology may become a future research hotspot. We hope that this Research Topic will inspire new research and well deserve discussion, and we look forward to publishing future advances in this important area of neuropharmacology.
